# Dating the diversification of the major lineages of Passeriformes (Aves)

**DOI:** 10.1186/1471-2148-14-8

**Published:** 2014-01-15

**Authors:** Per GP Ericson, Seraina Klopfstein, Martin Irestedt, Jacqueline MT Nguyen, Johan AA Nylander

**Affiliations:** 1 Department of Zoology, Swedish Museum of Natural History, Box 50007, SE–10405 Stockholm, Sweden; 2 Department of Biodiversity and Genetics, Swedish Museum of Natural History, Box 50007, SE–10405 Stockholm, Sweden; 3 School of Biological, Earth and Environmental Sciences, University of New South Wales, Sydney NSW 2052, Australia; 4 BILS – Bioinformatics Infrastructure for Life Sciences, University of Linköping, SE–58183 Linköping, Sweden

**Keywords:** Passeriformes, Molecular dating, Fossil calibrations, New Zealand–Antarctica vicariance

## Abstract

**Background:**

The avian Order Passeriformes is an enormously species-rich group, which comprises almost 60% of all living bird species. This diverse order is believed to have originated before the break-up of Gondwana in the late Cretaceous. However, previous molecular dating studies have relied heavily on the geological split between New Zealand and Antarctica, assumed to have occurred 85–82 Mya, for calibrating the molecular clock and might thus be circular in their argument.

**Results:**

This study provides a time-scale for the evolution of the major clades of passerines using seven nuclear markers, five taxonomically well-determined passerine fossils, and an updated interpretation of the New Zealand split from Antarctica 85–52 Mya in a Bayesian relaxed-clock approach. We also assess how different interpretations of the New Zealand–Antarctica vicariance event influence our age estimates. Our results suggest that the diversification of Passeriformes began in the late Cretaceous or early Cenozoic. Removing the root calibration for the New Zealand–Antarctica vicariance event (85–52 Mya) dramatically increases the 95% credibility intervals and leads to unrealistically old age estimates. We assess the individual characteristics of the seven nuclear genes analyzed in our study. Our analyses provide estimates of divergence times for the major groups of passerines, which can be used as secondary calibration points in future molecular studies.

**Conclusions:**

Our analysis takes recent paleontological and geological findings into account and provides the best estimate of the passerine evolutionary time-scale currently available. This time-scale provides a temporal framework for further biogeographical, ecological, and co-evolutionary studies of the largest bird radiation, and adds to the growing support for a Cretaceous origin of Passeriformes.

## Background

Passeriformes (passerines) is the largest and most diverse avian order, comprising about 5,700 species and representing almost 60% of all living birds. Due to their ubiquity and enormous diversity, passerines have been the focus of many ecological, behavioral, anatomical and evolutionary studies. The systematic relationships, early evolution and biogeography of passerines have long been debated among avian taxonomists. While disagreeing on many other aspects of passerine systematics and evolution, most ornithologists of the 20th century agreed that the Passeriformes is one of the youngest avian orders. Therefore, it was rather surprising when molecular data proposed that the evolutionary history of these birds dated back to the end of the Cretaceous [1,2]. Under the current paradigm, passerines arose on the southern supercontinent Gondwana and major passerine lineages became isolated with the continental break-up. This hypothesis rests on an interpretation of the modern distributions of the major passerine clades: the New World suboscines in South and Central America (a dispersal to North America seems to have been facilitated by the formation of the Isthmus of Panama 3 Mya), the Old World suboscines in tropical Africa and Asia (except *Sapayoa aenigma*, which occurs in west Colombia), and all basal members of the oscines in the Australo–Papuan region. Furthermore, the endemic New Zealand wrens (Acanthisittidae) form the sister group to all other passerines. It has been postulated that the ancestors of the New Zealand wrens became isolated when the Zealandia continental fragment separated from Antarctica 85–82 Mya [3,4].

The supposed role of Gondwana in the diversification of passerines implies a late Cretaceous origin of this radiation. Several studies have used the 85–82 Mya date for the New Zealand–Antarctica vicariance event to calibrate estimates of the passerine evolutionary time-scale, typically in the absence of suitable fossils. There are two problems with this calibration method. First, it builds on the assumption that the New Zealand wrens were actually present on Zealandia when it separated from Antarctica. Under this assumption, the argument for a Cretaceous origin of passerines becomes circular. Second, it has been suggested that the last land connection is considerably younger than the postulated 85–82 Mya date that has been used to calibrate the passerine tree. Recent geological data suggest that the separation of New Zealand began about 85 mya but was not complete until about 55–52 mya [5-7]. Dispersal possibilities for terrestrial organisms may even have existed as late as the Paleogene [8]. Some geologists have argued that the entire landmass of New Zealand was completely submerged during the marine transgression in the Oligocene [9,10]. Phylogeographic and paleontological data, however, suggest that endemic terrestrial biota were present in the Oligocene, even though New Zealand was reduced to a few scattered islands or an estimated 18% of the current landmass [11,12] and papers cited therein].

The purpose of this paper is two-fold. First, we aim to provide an updated time-scale for the evolution of the major passerine clades. Second, we aim to present age estimates for major groups of passerines, which can be used as secondary calibration points in future phylogenetic studies of passerines at lower taxonomic levels.

Our estimates of the passerine phylogeny and evolutionary time-scale are based on 7,193 bp obtained from seven nuclear genes. We calibrate the molecular clock using both fossils and the New Zealand–Antarctica vicariance event described above, but with an updated interpretation of the time frame of this geological event [5-7]. Unlike many previous analyses, we also incorporate the uncertainty in these calibrations, and assess the sensitivity of our age estimates to different interpretations of the New Zealand–Antarctica vicariance event.

## Methods

### Selection of taxa and gene sequences

The ingroup consists of 55 taxa and includes representatives of almost all of the basal lineages of passerines identified in previous molecular phylogenies (e.g., [13]). Most of the sequence data are from Alström et al. [14], Barker et al. [1,13], Ericson et al. [2,15,16], Ericson and Johansson [17], Fjeldså et al. [18,19], Fuchs et al. [20,21], Gelang et al. [22], Irestedt et al. [23-25], Irestedt and Ohlson [26], Johansson et al. [27], Jønsson et al. [28], Ohlson et al. [29], Zuccon and Ericson [30] and Zuccon et al. [31]. We have also added 25 novel sequences to the data set, which we have deposited in GenBank (Additional file 1: Table S1). Although some lineages are represented in the analyses by sequences obtained from different individuals, the concatenated sequences are in most cases from conspecific or congeneric individuals (Additional file 1: Table S1). Only for the Meliphagidae, Callaeidae and Petroicidae were we forced to combine sequences from more than one genus.

The total alignment consists of 7,193 bp obtained from seven genes: *MOS*, oocyte maturation factor mos (622 bp in the alignment/177 parsimony-informative characters), *MYC*, c-myc proto-oncogene exon 3 (504/83 bp), *GAPDH*, glyceraldehyde-3-phosphodehydrogenase intron 11 (419/222 bp), *MB*, myoglobin intron 2 (800/299 bp), *ODC1*, ornithine decarboxylase introns 6–7 (749/316 bp), *RAG1*, recombination activating protein 1 (2 947/740 bp), and *RAG2*, recombination activating protein 2 (1 152/326 bp). Details of extraction, amplification and sequencing procedures are given for *MOS* by Cooper and Penny [32], for *MYC* by Irestedt et al. [23], for *MB* by Irestedt et al. [33], for *GAPDH* by Fjeldså et al. [18], and for *ODC1* by Allen and Omland [34].

### Bayesian phylogenetic inference

For Bayesian inference of phylogeny and divergence times, the program MrBayes v.3.2 [35] was used to obtain Markov Chain Monte Carlo (MCMC) approximations of posterior tree distributions. Gene partitions were analyzed both separately and in concatenation. In the concatenated analyses, the dataset was partitioned by gene and by codon position (combined first and second versus third codon positions). Nucleotide substitution models were unlinked across partitions, and a reversible-jump MCMC over the space of all GTR sub-models was run for each of them (“nst = mixed” command in MrBayes [36]). Among-site rate variation was modeled using a discrete gamma distribution with four categories and a proportion of invariant sites [37]. We used partition-specific rate multipliers with a Dirichlet-distributed prior to allow the overall evolutionary rates to differ among partitions.

For all analyses, four Metropolis-coupled chains (temperature constant set to 0.1) were run for a minimum of 30 million generations, sampling every 1,000th generation. Four independent runs for the final analyses were conducted with the preferred clock model (see below), whereas two runs were done for the remaining analyses. Parameter and tree files were analyzed using Tracer [38] and AWTY [39] to check for convergence issues and suitable burn-in. Average standard deviations of split frequencies fell below the recommended value of 0.01 after about 5 million generations, indicating good topology convergence. After being scrutinized for convergence, tree and parameter files from separate runs were then concatenated to maximize precision. Calculations of credibility intervals, statistical analyses, and graphical output were generated using the R statistical programming language [40], utilizing functions in R-packages APE [41], PHANGORN [42], and PHYLOCH [43].

### Clock and tree model choice

We used stepping-stone sampling [44] to obtain estimates of the marginal likelihoods to choose between different clock models: the strict clock, a relaxed, auto-correlated log-normal clock (TK02 [45]), and a relaxed, uncorrelated gamma clock model (the white-noise or independent gamma rates (IGR) model [46]). We considered convergence to be acceptable when the standard deviation of split frequencies was below 0.04 in each of the steps. To achieve this, we ran 100,000,000 generations in total, distributed over 49 steps between the posterior and the prior, treating the first step as a burn-in. Within each step, 500,000 generations were discarded as a burn-in, and samples were drawn every 1,000 generations over the remaining 1,500,000 generations. Bayes factor comparisons strongly preferred the uncorrelated gamma relaxed clock model over the auto-correlated and strict clock models (2* ln(Bayes factor) equals 14.3 and 132.5, respectively [47]), and we used this model for subsequent analyses. In order to enhance convergence of the clock rate as well as tree height, we followed an empirical Bayes approach [48]. We initially ran a strict-clock analysis without calibrations in order to obtain an estimate for the tree height in substitution units. This value was then divided by the average tree height in million years according to the prior on the root (see below) in order to get a first estimate of the clock rate. This estimate was used as the median for a log-normal prior with a large standard deviation of 1, leading to an only weakly informative prior. For the priors on the variance of the relaxed-clock models, we used an exponential (10) prior.

Instead of relying on explicit models of the branching process to obtain a prior for the tree, we used the recently proposed extension of the uniform tree prior to calibrated clock settings [48]. Very little is known about the relative performance of different tree priors (with one notable exception [46]), but given the incomplete and uneven taxon sampling in our dataset, we preferred to use the uniform prior which is rather uninformative. Furthermore, it does not require the setting of additional parameters as in the birth-death or coalescent priors, and does not require any assumptions to be made about the sampling strategy.

### Calibration of the rate of evolution

To calculate absolute ages from the trees, two categories of calibration points can be used: geological vicariance events and fossils. The only geological vicariance event used in our analysis is based on the assumption that the New Zealand wrens became isolated when this continental fragment separated from Antarctica. However, it is now currently understood that separation of New Zealand was not complete until about 55–52 Mya [5-8,49]. In addition to using this best current estimate in the form of a uniform prior on the root age, we tested the previously used range of 85–82 Mya [50] and a *de facto* removal of the root calibration (uniform prior between 4,000 and 20 Mya).

**Figure 1:**
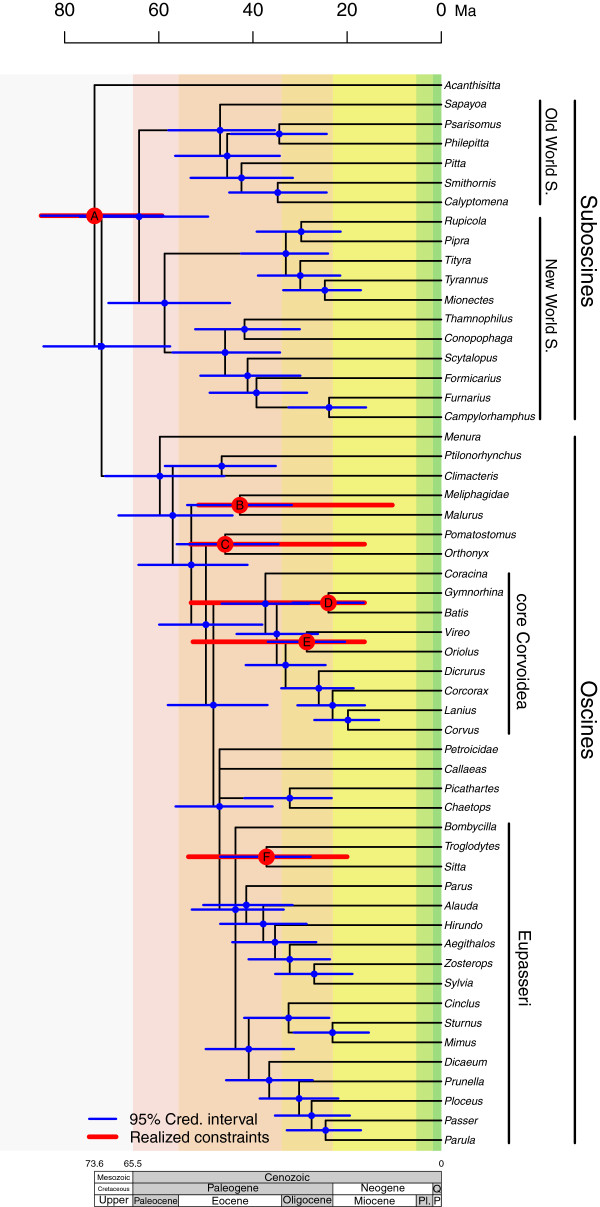
**Phylogenetic estimate of the evolutionary time-scale of passerines, with 95% credibility intervals plotted for estimated divergence times (thin blue bars).** Thick red bars represent the realized age priors (95%) for the constrained nodes. Labels **A-F** indicate calibration points and fossil placements, **A**: vicariance event separating New Zealand from Antarctica, **B**: honeyeater (Meliphagidae) [44], **C**: logrunner (Orthonychidae) (JMTN, unpublished observations), **D**: crown-group cracticid (Cracticidae) [41], **E**: oriolid (Oriolidae) [42], **F**: "climbing Certhioidea" [43]. Panel below figure indicates the geological time line; Q: Quaternary, Pl.: Pliocene, P: Pleistocene.

There are currently extremely few Cenozoic fossils of passerines that can be unambiguously assigned to recent lineages and can thus serve as calibration points in this study. We used three representatives of crown group passerines that come from Early Miocene (16.3–23 Mya) deposits in Australia, and their placement in our chronogram is given in Figure 1. They were identified as a logrunner (Orthonychidae) (JMTN, unpublished observations) (clade C in Figure 1), a crown-group cracticid (Cracticidae) [51] (clade D in Figure 1), and an oriolid (Oriolidae) [52] (clade E in Figure 1). Another fossil useful for calibration is a tarsometatarsus from the Early Miocene (MN 3, 20.5–18 Mya) of Germany, assigned to the “climbing Certhioidea”, a clade comprising treecreepers (Certhiidae), nuthatches and wallcreepers (Sittidae) [53] (clade F in Figure 1). In addition to these fossils, we used a calibration based on a fossil honeyeater (Meliphagidae) from the Middle Miocene of Australia (10.4–16.3 Mya) [54] (clade B in Figure 1). These fossils provide minimum age for their respective lineages, and we used offset exponential distributions as priors on the five calibrated nodes. Means for the prior distributions were obtained from Ericson et al. [55], using the estimated divergence between Tyrannidae and Laniidae (58 Mya) as a mean for all fossil calibrations within Passeriformes. This places most of the prior probability on younger ages, but also allows for older estimates. Because realized marginal prior densities of calibrated nodes can deviate substantially from the specified priors when multiple calibrations are used [56], we estimated the joint prior density of node ages by running an MCMC without data (by utilizing the “mcmc data = no” option in MrBayes). These realized priors are given in Figure 1.

Presence of the six calibrated nodes was enforced using monophyly constraints in the dating analyses. These nodes had high support in a previous, uncalibrated and unconstrained analysis (see electronic supplementary material). In addition to the nodes used for calibration, we invoked a rooting constraint between *Acanthisitta* and the remaining passerines. This was because a preliminary, uncalibrated relaxed-clock analysis showed the clock model to be insufficient to recover the correct root [35,57].

## Results

### Phylogenetic relationships

The Bayesian analysis using an uncorrelated gamma relaxed clock model (IGR) produced a phylogenetic estimate that agrees strongly with that obtained from an analysis without a clock and with previous studies based on nuclear DNA data (e.g., [13,58]) (Figure 1, for node numbers see Additional file 2: Figure S1). Most clades in the tree have high posterior probabilities (Additional file 3: Table S2), with the exceptions occurring in the same parts of the tree that have previously been difficult to resolve.

The tree was rooted with the New Zealand wrens (Acanthisittidae), with the remaining taxa forming two clades, the suboscines and the oscines. The suboscines are divided into two reciprocally monophyletic clades consisting of taxa distributed in the New World and Old World, respectively. The New World suboscines are further divided into Furnariida and Tyrannida. Within each of these groups the inferred phylogenetic relationships agree well with those from previous studies (e.g., [29,59-62]).

Within the oscines, the delimitation of Passerida has been much discussed [13,17,26,27]. The question concerns the relationships between Eupasseri (the core group of Passerida) and the five families Picathartidae (rockfowl), Chaetopidae (rockjumpers), Eupetidae (rail-babblers) (not included in this study), Petroicidae (Australasian robins) and Callaeidae (New Zealand wattlebirds). With the addition of more nuclear markers, Picathartidae and Chaetopidae form a sister pair that is placed in an unresolved clade with Petroicidae, Callaeidae and Eupasseri. This tree topology does not contradict the hypothesis that the insertion of one amino acid in *MYC* in Picathartidae, Chaetopidae, Callaeidae and Eupasseri, but not in Petroicidae, may be a synapomorphy for Passerida [17,63]. The well-supported parts of the inferred relationships within Passerida all agree with previous phylogenetic studies of this radiation (e.g., [13,17]).

### Sensitivity of divergence-time estimates to the root prior

The prior specified for the root age had a considerable effect on the posterior of the age of the passerine radiation. As expected, the 95% credibility intervals were narrowest when the most restrictive prior was used, i.e., a uniform distribution between 85 and 82 Mya for the root node (posterior: median = 83.5, 95% credibility interval = 85.0–82.2 Mya). Widening the prior interval to 85–52 Mya, which better reflects our current understanding of the geological events leading to the separation of New Zealand from Antarctica [5-7], we obtained a posterior median around 73.6 Mya (85.0–59.0 Mya). When *de facto* removing the root calibration, the median estimate increased to 90.8 Mya, but with a very large uncertainty (125.6–49.4 Mya).

### Performance of individual genes for divergence-time estimation

**Figure 2:**
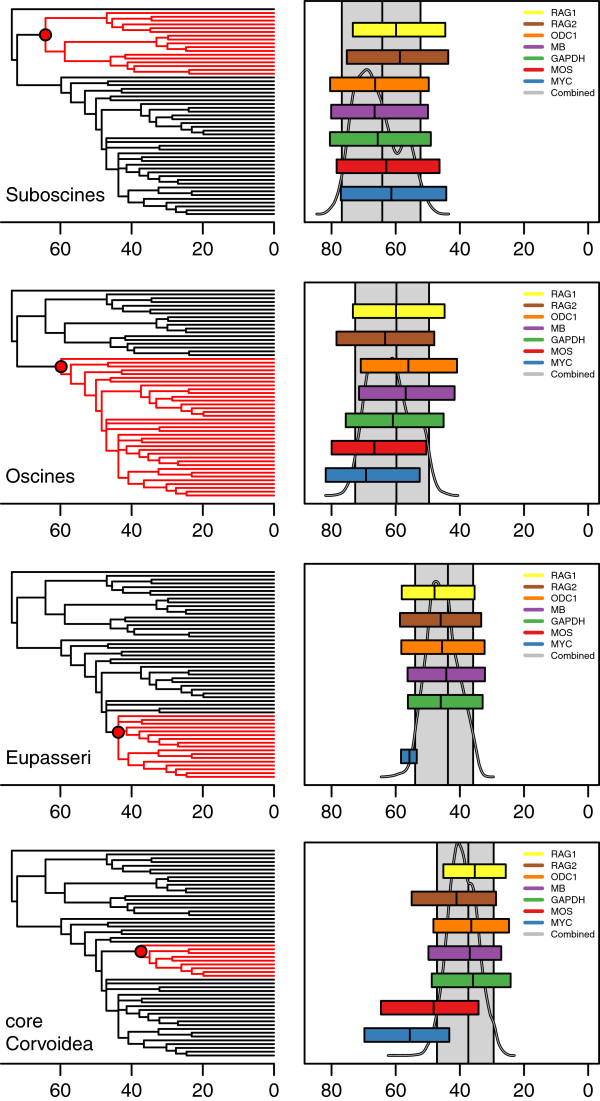
**Divergence-time estimates (95% credibility intervals) from individual genes (colored boxes) compared with the concatenated gene analysis (95% credibility intervals, gray background box, and posterior density, gray line) for selected groups.** Genes are ordered top-down by the number of parsimony-informative characters.

Divergence-time estimates for the clades in the consensus tree based on the combined data are given in Figure 1, and Additional file 3: Table S2. The 95% credibility intervals for individual gene estimates are given in Figure 2, where they are compared with the estimates from the combined data analysis. Intervals for certain genes may be missing at some nodes because these nodes were not recovered in the individual gene trees, which provides insights into gene incongruence. The trees inferred from both *ODC1* and *RAG2* were fully congruent with that from the combined analysis. In contrast, the remaining gene trees showed some topological incongruence, with *GAPDH* and *MB* differing in only one clade and *RAG1* in two. *MOS* and *MYC* showed the highest topological incongruence, with four and five nodes missing, respectively. The incongruence seems to be concentrated in the regions of low resolution and within the period 47–36 Mya (Figure 1, Additional file 3: Table S2).

**Figure 3:**
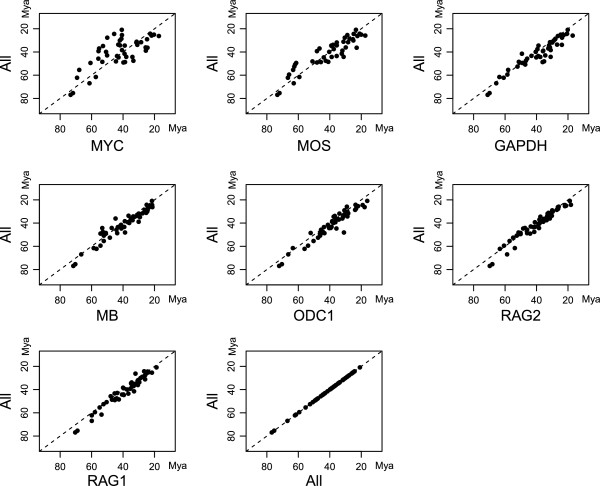
**Median divergence-time estimates from individual genes (x axes) versus the combined data (y axes, “All”) for corresponding nodes in the tree from the analysis of the combined data.** The dotted line represents the one-to-one correlation. Genes are ordered from left to right and top-down by the number of parsimony-informative characters. The last, trivial, correlation (All vs. All) is included to illustrate the concentration of node estimates over time.

To summarize the performance of each individual gene, we examined the correlation between the divergence-time estimates from individual genes and from the combined analysis (Figure 3). The gene with the least amount of data (*MYC*) also displays the largest variation and deviance from the combined analysis (Kendall's τ = 0.45, p < < 0.01). All other genes produce estimates that are in better accordance with the combined estimates, with τ ranging from 0.746 (*MOS*) to 0.85 and 0.90, for *RAG1* and *RAG2*, respectively. These two genes were probably the most influential in the combined estimation. The shorter genes tended to yield overestimates of ages, especially for nodes older than around 40 Mya, but underestimated the ages of younger nodes compared to the combined analysis (Figure 3).

**Figure 4:**
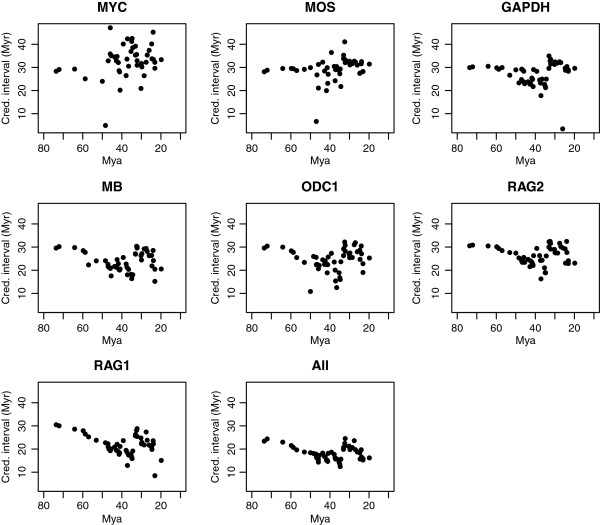
**Widths of the 95% credibility intervals of divergence-time estimates for individual genes over time.** Genes are ordered from left to right and top-down by the number of parsimony-informative characters.

There is a considerable variation in the precision (width of 95% credibility intervals of age estimates), both among genes and for different nodes (Figure 4). There is a significant difference in the widths of the 95% credibility intervals among genes (Kruskal-Wallis, χ^2^ = 75.62, p < < 0.01), ranging from the longest (*MYC*) with a mean width of 12.0 My, to the shortest (*RAG1*) with a mean width of 8.2 My. In comparison, the combined analysis yielded age estimates with a mean 95% credibility interval width of 7.0 My.

An alternative way of examining congruency of estimates among genes, in combination with precision, is to evaluate the extent of overlap in 95% credibility intervals for individual nodes among genes (Additional file 4: Figure S2). Despite the large variation observed, there is a fairly good overlap between individual gene estimates. For some nodes, both congruence and precision are high for the majority of genes.

## Discussion

### Phylogenetic relationships

The tree topology inferred from the Bayesian analysis of the combined data agrees well with previous estimates based on multiple nuclear and mitochondrial markers. The taxa that are difficult to place phylogenetically in the present analysis have also been problematic in other studies. Increasing the number of nuclear genes in the data set did not help to resolve the relative positions of *Pitta*–*Calyptomena*–*Smithornis* cf. [24], *Pipra*–*Rupicola* cf. [29,61,64], and Petroicidae vs. *Picathartes*–*Chaetops* cf. [26,28,65]. Furthermore, as in previous analyses (e.g., [1,13,17,66]), *Parus* and *Bombycilla* cannot be confidently placed. This suggests that genome-scale sequence data are necessary to resolve these splits, or that the diversification in certain parts of the tree occurred too rapidly to be resolved with confidence.

### Utility of individual genes for divergence-time estimates

The factors determining the utility of a gene for topological inference have been investigated in several studies (e.g., [67-73]), but there has been less attention on branch-length estimation [74]. However, the latter is crucial for understanding the performance of different genes in estimating node ages. In our analyses, we found considerable differences among genes in their ability to provide congruent (Figure 3) and precise (Figure 4) divergence-time estimates. These gene-specific performances also varied over branches in the tree (Additional file 4: Figure S2).

One factor that is likely to affect a gene's performance is the size of the data set. As the size of the data set grows, sampling error and the width of the 95% credibility intervals will tend to decrease (down to a limit set by the prior [75]). This improvement in precision with an increased amount of data is apparent in our analyses (Figure 4, Additional file 3: Figure S1). Interestingly, lower precision seemed to be confined to a particular time period in the tree (ca. Oligocene; Figure 1). The uncertainty in divergence-time estimates for this time period affected all genes, which is inconsistent with the notion that “some genes are better” for different ages.

Another factor that might affect performance of a gene is its substitution rate. A method for predicting the utility of a gene is to examine its saturation levels. A saturated gene would reach a plateau in pair-wise sequence distances over time. Using this approach, we did not find pronounced saturation in any of the individual genes or the combined data (Additional file 5: Figure S3). As an example, the *MYC* gene, which seems to be performing less well than other genes, is not the most rapidly evolving among the genes included in our study, but has genetic distances similar to the combined data. Hence, saturation, on its own, does not seem to be a good predictor of gene performance.

Other factors that can affect the performance are clock- and/or substitution-model inadequacy. The models used in the analyses might fail to capture the actual patterns of rate variation and correctly estimate branch lengths and divergence times. This might explain the tendency to over- or underestimate divergence times at different depths in the tree (Figure 3).

Finally, different genes might support different topologies (gene trees), making them less congruent or precise when compared to a concatenated tree (“species tree”). In this study, we assume that the gene trees evolved on the same topology by concatenating all of the genes in a single analysis. However, we could quantify the incongruence by looking at gene-specific estimates and identifying regions of conflict (Additional file 4: Figure S2). This approach allows us to focus on clades for which dates were estimated with high precision, and where the majority of the genes have high overlap in their 95% credibility intervals.

### Calibrating the passerine phylogeny

The increased availability of molecular data allows us to estimate phylogenetic relationships and relative branch lengths with unprecedented precision, but dating analyses still mostly depend on how the molecular clock is calibrated [76,77]. While novel approaches that directly use fossil data are certainly preferable [35,78], they require high-quality morphological datasets for both fossil and extant taxa which are currently not available for most groups. Divergence-time estimates thus still largely rely on an adequate translation of the fossil record or of certain vicariance hypotheses into priors for node ages [79].

Our study took a combined approach, using available relevant passerine fossils in combination with an interpretation of the New Zealand-Antarctica vicariance event that is in better accordance with the most recent geological evidence [5-7]. We also used two additional root calibrations to assess sensitivity. The resulting age estimates were sensitive to the vicariance calibration, an outcome that could be expected for a calibration at the root. Additionally, calibrations based on vicariance events, in contrast to fossils, also provide a maximum age constraint [78]. Removing the root calibration altogether led to inflated 95% credibility intervals and older median ages, indicating that the mean used for the fossil calibrations (obtained from 55, see Methods) was not sufficient to provide upper bounds on the node ages. In contrast, when we used the traditional, narrow interpretation of the vicariance event, i.e., an age of the *Acanthisitta* split of 85–82 Mya, we obtained much more precise age estimates for the passerine radiation. When widening the prior to 85–52 Mya, which more closely matches the current interpretation of the history of the break-up of eastern Gondwana [5-8], divergence-time estimates became somewhat less precise, but younger. Given that divergence-time estimates relying on a narrow interpretation of the New Zealand vicariance event are probably biased, we have focused on the results obtained using the more relaxed prior on the age of the passerine root. By using both the relevant passerine fossils and an up-to-date interpretation of the New Zealand–Antarctica vicariance event, our analysis arguably provides the best estimate of the passerine evolutionary time scale.

### Divergence times between major groups of Passerines

Our estimated dates of divergences (Figure 1) are generally younger than those from previous studies based on a large sample of passerine species (e.g. [13], see also [80]). Barker et al. [13] used a fixed age (82 Mya) for the split between New Zealand and Antarctica as a single calibration point for the passerine tree, which may explain the difference between the estimated divergence times compared to our study.

The mean divergence times in the basal part of the tree are all late Cretaceous in age. For example, the split between suboscines and oscines is estimated at 71.4 Mya (84–58 Mya) (Table 1, column A). This is considerably younger than the 91.8 Mya estimated by Pereira and Baker [81], and slightly younger than the two estimates (77.4 and 76.8 Mya) obtained with different dating methods by Barker et al. [13] (Table 1, columns D and E). Unlike our study and that of Barker et al. [13], Pereira and Baker [81] studied mitochondrial data (whole genomes). Their study also had a broader taxonomic scope across birds, with passerines represented only by one suboscine species and one oscine species. In our study, as well as those of Barker et al. [13] and Pereira and Baker [81], the divergence between suboscines and oscines is estimated to have occurred in the late Cretaceous. This is in agreement with the postulated role of the break-up of Gondwana in the diversification of passerine birds [1,2].

**Table 1:** Comparison of divergences times for five major clades of passerines as estimated in our study and in previous studies

	**A: Present study**		**B: Present study**		**C: Pereira & Baker (2006)**		**D: Barker et al. (2004)**		**E: Barker et al. (2004)**		**F: Ericson et al. (2002)**	
	**Time (mya)**	**CI**	**Time (mya)**	**CI**	**Time (mya)**	**CI**	**Time (mya)**	**CI**	**Time (mya)**	**CI**	**Time (mya)**	**CI**
Split Suboscines vs. Oscines (node 2)	72.1 (71.4)	84 – 58	89.0 (88.6)	123 – 49	91.8	103 – 81	77.4	81 – 73	76.8	80 – 72	71.0	79 – 62
Split OW vs. NW Suboscines (node 35)	64.2 (63.6)	77 – 50	79.2 (78.8)	110 – 44	–	–	70.5	78 – 65	68.8	73 – 61	70.5	78 – 65
Oscines (node 3)	59.8 (59.4)	71 – 46	73.6 (73.5)	104 – 41	–	–	64.7	70 – 60	63.1	67 – 57	64.7	70 – 60
Core Corvoidea (node 24)	37.4 (37.4)	47 – 28	45.3 (45.8)	65 – 25	–	–	40.4	45 – 35	38.6	42 – 32	40.4	45 – 35
Eupasseri (node 9)	43.7 (43.5)	53 – 33	53.6 (53.5)	74 – 30	–	–	40.7	54 – 15	40.8	49 – 36	40.7	54 – 15
DNA data	Seven nuclear genes (7,204 bp)		Seven nuclear genes (7,204 bp)		Complete mtDNA		Two nuclear genes (4,126 bp)		Two nuclear genes (4,126 bp)		Two nuclear genes (1,407 bp)	
Calibration(s)	Five passerine fossils and one vicariance event, split between New Zealand and Antarctica (85–52 mya),		Five passerine fossils and essentially no time-constraint for New Zealand and Antarctica vicariance event (4,000–20 Mya)		Thirteen fossils, of which five avian (no passerine)		One vicariance event, split between New Zealand and Antarctica (82 mya)		One vicariance event, split between New Zealand and Antarctica (82 mya)		One vicariance event, split between New Zealand and Antarctica (82 mya)	
Dating method (program)	IGR relaxed clock, Bayesian inference (MrBayes 3.2)		IGR relaxed clock, Bayesian inference (MrBayes 3.2)		Bayesian rate autocorrelation (Multidivtime)		Penalized likelihood (r8s)		Nonparametric rate smoothing (r8s)		Local rates method (Rhino)	

CI = 95% confidence or credibility interval, respectively.

Estimated dates of divergences with 95% credibility intervals for five major clades of passerines as obtained in our study under different root priors (columns A and B) and in previous studies (columns C to F). The dates of divergences estimated herein are generally younger than those previously published in studies that have included a large sample of passerines. Node numbers refer to Additional file 2: Figure S1 and Additional file 3: Table S2.

Within the suboscines, the Old World and New World taxa form two monophyletic clades, with the exception of the Colombian species *Sapayoa aenigma* (Broad-billed Sapayoa) which has been shown to belong to the Old World clade [18]. It has been speculated that the ancestors of the New World suboscines became isolated in South America when it split from Antarctica around the end of the Cretaceous [2]. The ancestor of the Old World suboscines may have colonized the African and Indian land masses (at this time already separated from Antarctica) from the West Antarctic Peninsula via island chains on the Kerguelen and Crozet Plateaus (along the Mascarene, Maldive and Ninety East Ridges) [18]. The mean date for the split between the suboscines in the Old World and New World is estimated at 63.6 Mya (77–50 Mya) (Table 1, column A). This is 5–7 My younger than previous estimates [13] (Table 1, columns D and E), which raises doubts about the biogeographic scenario suggested for the dispersal of the Old World suboscines from Antarctica to Africa and India.

Some biogeographic analyses have suggested that the oscines evolved in Australia after its split from Antarctica [1,2]. In our study, the mean date estimate for the earliest split in extant oscines is 59.4 Mya (71–46 Mya). Previous estimates by Barker et al. [13] are 3–5 My older (Table 1, columns D and E). The core Corvoidea clade began to radiate at 37.4 Mya (47–28 Mya) according to our estimates, compared to 40.4 and 38.6 Mya, as estimated by Barker et al. [13] using two different algorithms.

Eupasseri consists of all species of Passerida except Petroicidae, Picathartidae, Eupetidae and Callaeidae, all of which systematists at some point have included in Passerida (e.g., [82] and papers cited therein). We estimate the age of Eupasseri at 43.5 Mya (53–33 Mya), which is only 1–3 My younger than the estimates by Barker et al. [13] (Table 1, columns D and E).

The hypothesis of a Cretaceous origin for the passerine radiation is controversial, as this date is much older than would be expected from the fossil record [83-85]. The main criticism is the use of the New Zealand–Antarctica vicariance event for calibrating the tree. The rationale for using this calibration is the distinct Gondwanan signature of the passerine phylogeny [80,86], with the basal clades distributed in different southern continents. However, both overall phylogenetic patterns and molecular clock estimates support an old age of the passerine radiation. By applying the passerine evolutionary rate for mitochondrial genes [87], Barker et al. [13] estimated the age of the split between the New Zealand wrens and all other passerines at 87 Mya. Our data support a Cretaceous origin of Passeriformes when we apply recent estimates of gene-specific evolutionary rates by Lerner et al. [86] to nuclear genes analyzed in our study. Lerner et al. [88] used island ages to obtain a dated phylogeny and rates of evolution for a number of genes in a study of Hawaiian honeycreepers. Three of the genes examined in their study were also included in our analyses. For each gene, we estimated the root age using these rates and using the respective tree heights in substitutions from our main analysis (using the rate multiplier in MrBayes). Even though this yielded rather crude estimates, the overall patterns are consistent with those from our calibrated analysis (estimate from *RAG1*: 115.8 Mya, 137–97 Mya; *GAPDH*: 99.2 Mya, 119–81 Mya; *ODC1*: 56.2 Mya, 67–47 Mya).

### Divergence-time estimates suitable for use as secondary calibrations for studies of passerine evolution

Most phylogenetic analyses of passerines do not include *Acanthisitta*, which precludes the use of the split between New Zealand and Antarctica to calibrate the tree. Owing to the paucity of the passerine fossil record and the difficulty with which they can be taxonomically identified below suborder level, there are few fossil passerines that can provide useful calibrations for molecular studies of divergence times. Until our understanding of geological vicariance events improves and more fossils are discovered and described, we are confined to using secondary calibration points to estimate divergence times within the passerine radiation. The extent of overlap in 95% credibility intervals of age estimates from different genes gives an idea of the suitability of an individual node as a secondary calibration point. Despite the large variations in 95% credibility intervals between genes, there is a fairly good overlap between individual gene estimates for most nodes (Additional file 4: Figure S2). We find that several nodes receiving a high posterior probability (1.0) in the phylogeny presented here provide good candidates for secondary calibration points, particularly the suboscines, oscines, core Corvoidea and Eupasseri (Figure 2, Table 1). Divergence-time estimates from our combined dataset can be used in further molecular analyses of the passerine phylogeny.

### Implications of a Cretaceous age for the passerine radiation

Molecular data suggest that the earliest diversification of passerines took place in the late Cretaceous. In studies that use a narrow 85–82 Mya span for the New Zealand–Antarctica vicariance event to calibrate the passerine phylogeny [1,2], this conclusion is inevitably reached by default. However, we also inferred a late Cretaceous age for the earliest evolution of passerines even when widening the age range for the vicariance event to the more realistic 85–52 Mya span and including passerine fossils for calibration (Table 1, column A). Furthermore, when we removed the influence of the New Zealand–Antarctica vicariance event and relied solely on the fossils, the median age of the passerine root fell in the Cretaceous (Table 1, column B), albeit with a wide 95% credibility interval reaching well into the Cenozoic. Cretaceous ages for the earliest passerine diversifications are also implied when using independent estimates of substitution rates for individual genes. Barker et al. [13] showed this for the mitochondrial cytochrome b gene, and our analyses yield similar results for two out of the three nuclear genes that were calibrated in this manner.

Another observation consistent with a Cretaceous origin of the passerines is that their phylogenetic relationships reveal a biogeographic pattern that has a clear Gondwanan signature. The basal oscines, the New World suboscines and the Old World suboscines, are all confined to continents that were once part of Gondwana. Although passerines could easily have dispersed, obscuring their biogeographic patterns, birds surprisingly often exhibit strong biogeographic patterns that are closely linked to their evolutionary history. This is true regardless of whether we study phylogeographic patterns within a species or geographic distributions of families and other higher-level taxa. The current distributions of the basal oscines, New World suboscines and Old World suboscines may be important in revealing the earliest history of the passerines, and this may also be true for the current distribution of the New Zealand wrens.

A Cretaceous origin for the passerine radiation has far-reaching implications, as it suggests the fossil record is severely incomplete [83,84]. The oldest passerine fossils are currently known from the early Eocene (ca 55 Mya) of Australia [89], which may represent stem passerines [85]. The oldest fossils in the Northern Hemisphere date to the early Oligocene (34–32 Mya) of Europe (see [85] and references therein). These fossils include representatives thought to be outside crown group Eupasseres [90,91], a suboscine-like passerine [92], and nearly complete skeletons of passerines of unknown affinities [93-95]. The earliest fossil passerines that can be confidently assigned to an extant family are from the early Miocene, including a New Zealand wren [12] and the fossils used to calibrate the nodes in our analyses [51-53]. It is probable that much of the early diversification took place in the southern continents from where comparatively few fossil sites from that time period are known. For example, there is an absence of passerine fossils in avifaunas from late Cretaceous and Paleogene sites in South America, and there is a significant gap in the Australian fossil record between the early Eocene and late Oligocene.

Mayr [85] has remarked that a late Cretaceous age of the passerines makes this radiation unique among endothermic vertebrates in having remained morphologically “virtually unchanged for 80 million years”. This is a major reason for why he is skeptical of the suggested age for the group, in addition to the lack of Cretaceous fossils. Passerines are indeed a morphologically uniform group, but this is mostly true for body parts that do not directly relate to locomotion and feeding. In passerines, the morphology associated with locomotion and feeding has shown substantial evolutionary plasticity. Following the advent of DNA-based taxonomy, the literature has been filled with examples of groups that have evolved similar adaptations by convergence (e.g., [59]), which has misled avian systematists for hundreds of years. Unfortunately the fossil record of passerines is silent on this point because very little is known about the morphology associated with feeding and locomotion in the few Paleogene passerines found.

## Conclusions

In this study, we used a Bayesian relaxed-clock approach to estimate the evolutionary time-scale of the major clades of passerines based on seven nuclear genes, five passerine fossils, and an updated interpretation of the New Zealand split from Antarctica. We found no support from molecular data or from the overall biogeographic patterns of the passerines to refute the possibility of a Gondwanan origin, and thus a Cretaceous age, of the group. Our results represent the best estimate of the passerine evolutionary time-scale currently available and add to the growing support for the early Cenozoic diversification of this order. This temporal framework can be used in further biogeographical, ecological, and co-evolutionary studies of the Passeriformes. Furthermore, our analysis provides estimates of divergence times for major groups of passerines, which can be used as secondary calibration points in future molecular studies.

## Availability of data

The data sets supporting the results of this article are available in the GenBank (DNA sequences, accession nos. KF905607-KF905630) and TreeBASE (TreeBase link Study Accession URL: http://purl.org/phylo/treebase/phylows/study/TB2:S15185). Also see Additional file 6: text file S1 for MrBayes command files, including clock model settings and calibration information.

## Competing interests

The authors have no competing interests.

## Authors’ contributions

PE, SK, MI and JAAN designed the study. JAAN and SK performed the phylogenetic analyses, estimated divergence times and analyzed the data. MI did the lab work. JMTN provided fossil data for calibration. PE and JMTN evaluated potential fossil calibration points. PE, SK, MI and JAAN drafted the manuscript. All authors read, commented upon and approved the manuscript.

## Supplementary Material

Additional file 1: Table S1 Species names and GenBank accession numbers for sequences included in the phylogenetic analysis. First column lists the taxon names as they appear in the phylogeny. Taxonomy follows Dickinson EC (ed) 2003. *The Howard and Moore Complete Checklist of the Birds of the World.* Third edition. Princeton University Press, New Jersey. 1056 pp. Click here for file

Additional file 2: Figure S1 Chronogram inferred from the combined data (Figure 1), with node numbers corresponding to plots in Additional file 4: Figure S2. Click here for file

Additional file 3: Table S2 Divergence-time estimates for each node in the chronogram of the combined data set (Figures 1 and Additional file 2: Figure S1), along with posterior clade probabilities (PP), and proposals for "posterior as prior" specification to be used in secondary calibration. For the age estimates, the mean, median, lower and upper limits of the 95% credibility intervals (CI) are given. The priors are given as gamma (<mean>,<std.dev.>), centered using either the mean or the median of the posterior density. Node numbers refer to node labels in Additional file 2: Figure S1, and letters to labels in Figure 1. Click here for file

Additional file 4: Figure S2 Divergence-time estimates (median and 95% credibility intervals) from individual genes (colored boxes) compared with the concatenated gene analysis (median and 95% credibility intervals, gray background box). Nodes are ordered from oldest to most recent divergences, and node numbers correspond to node labels in Additional file 2: Figure S1 and Additional file 3: Table S2. A red frame indicates when a node is constrained with a calibrated node prior (see text). Click here for file

Additional file 5: Figure S3 Saturation plots for pair-wise sequence distances (uncorrected p-distance) over time (million years). Genes are ordered from left to right and top-down by the number of parsimony-informative characters. Click here for file

Additional file 6 **Text file S1.** MrBayes command files including clock model settings and calibration information. Click here for file
