# Supplementary figures and images for: Dating the diversification of the major lineages of Passeriformes (Aves)
Source: BMC Evol Biol. 2014 Jan 15;14:8. doi: 10.1186/1471-2148-14-8 (PMC3917694; doi:10.1186/1471-2148-14-8)

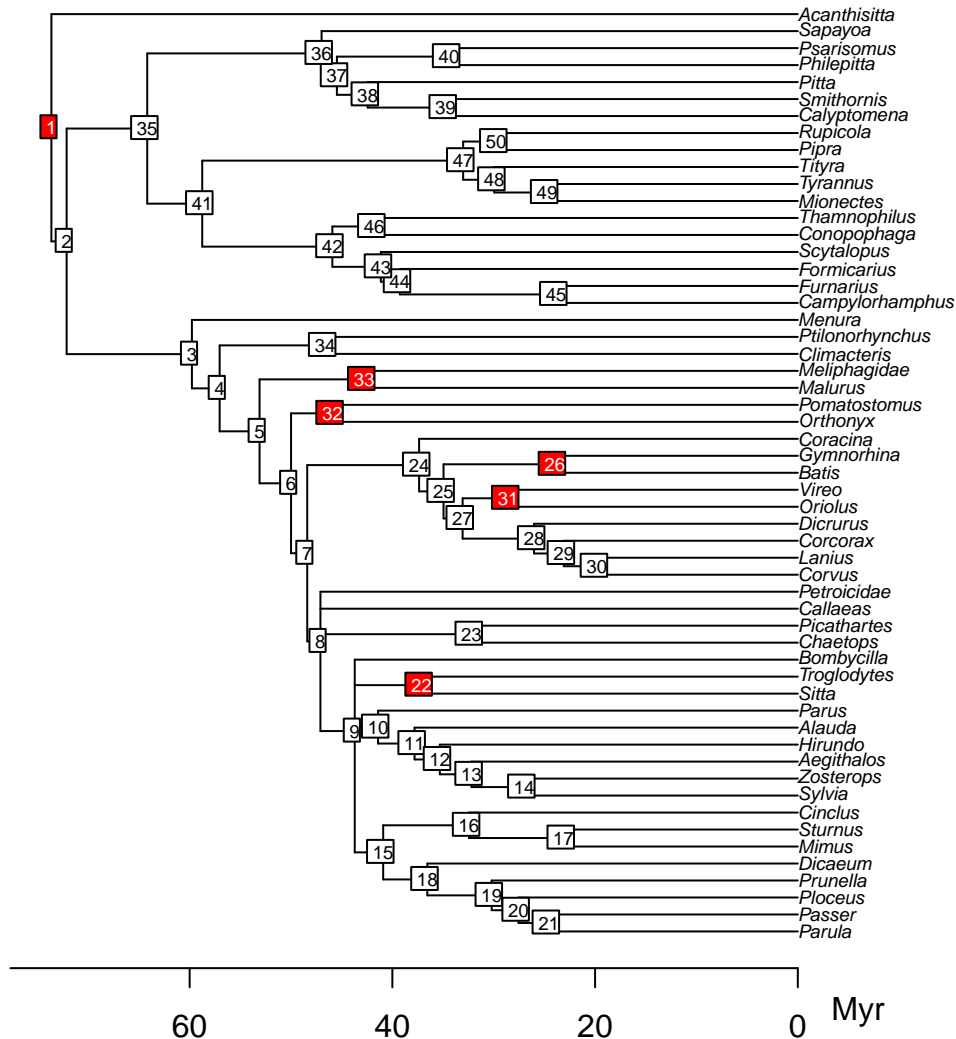

Supplement: Additional file 2: Figure S1 — Chronogram inferred from the combined data (Figure 1), with node numbers corresponding to plots in Additional file 4: Figure S2. [file 1471-2148-14-8-S2.pdf]

**MYC**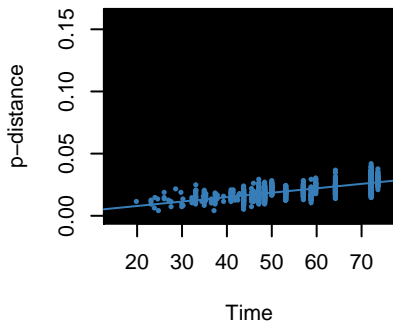**MOS**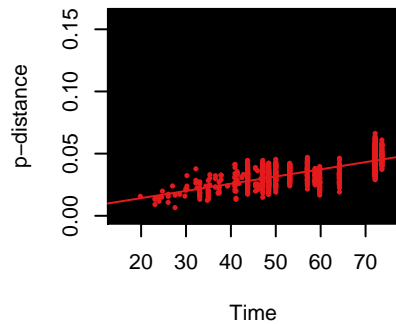**GAPDH**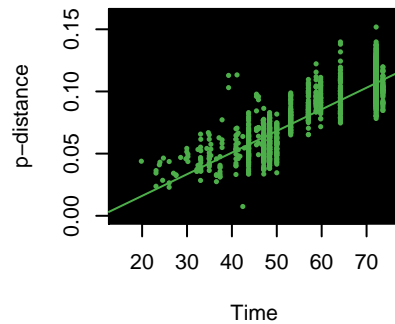**MB**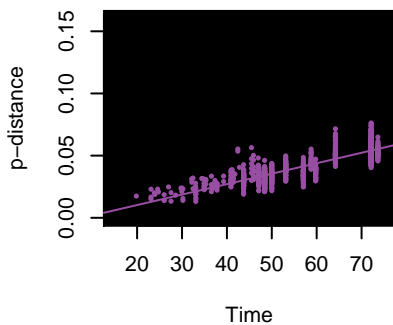**ODC1**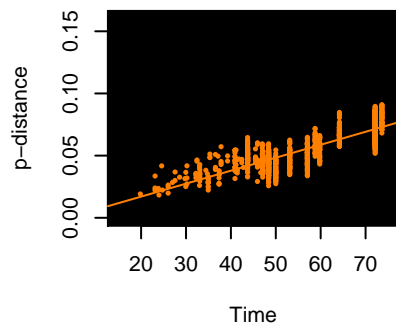**RAG2**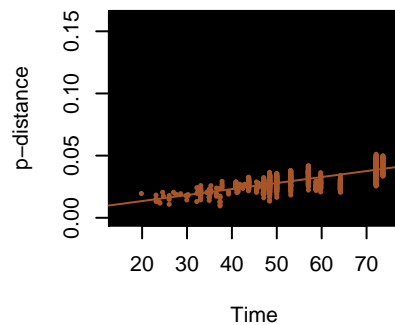**RAG1**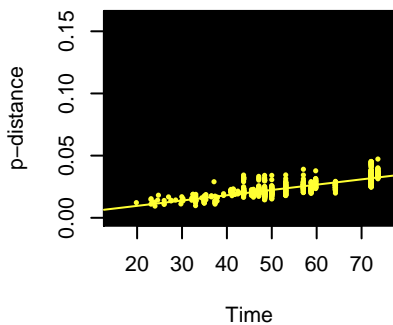**All**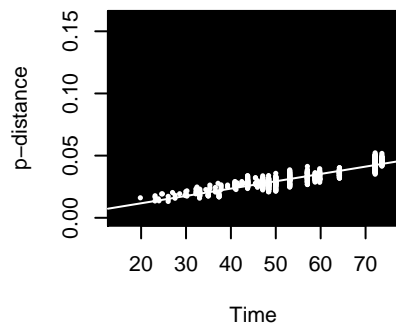

Supplement: Additional file 5: Figure S3 — Saturation plots for pair-wise sequence distances (uncorrected p-distance) over time (million years). Genes are ordered from left to right and top-down by the number of parsimony-informative characters. [file 1471-2148-14-8-S5.pdf]
