# Supplementary material for: Dating the diversification of the major lineages of Passeriformes (Aves)
Source: BMC Evol Biol. 2014 Jan 15;14:8. doi: 10.1186/1471-2148-14-8 (PMC3917694; doi:10.1186/1471-2148-14-8)
Supplement: Additional file 4: Figure S2 — Divergence-time estimates (median and 95% credibility intervals) from individual genes (colored boxes) compared with the concatenated gene analysis (median and 95% credibility intervals, gray background box). Nodes are ordered from oldest to most recent divergences, and node numbers correspond to node labels in Additional file 2: Figure S1 and Additional file 3: Table S2. A red frame indicates when a node is constrained with a calibrated node prior (see text). [file 1471-2148-14-8-S4.pdf]

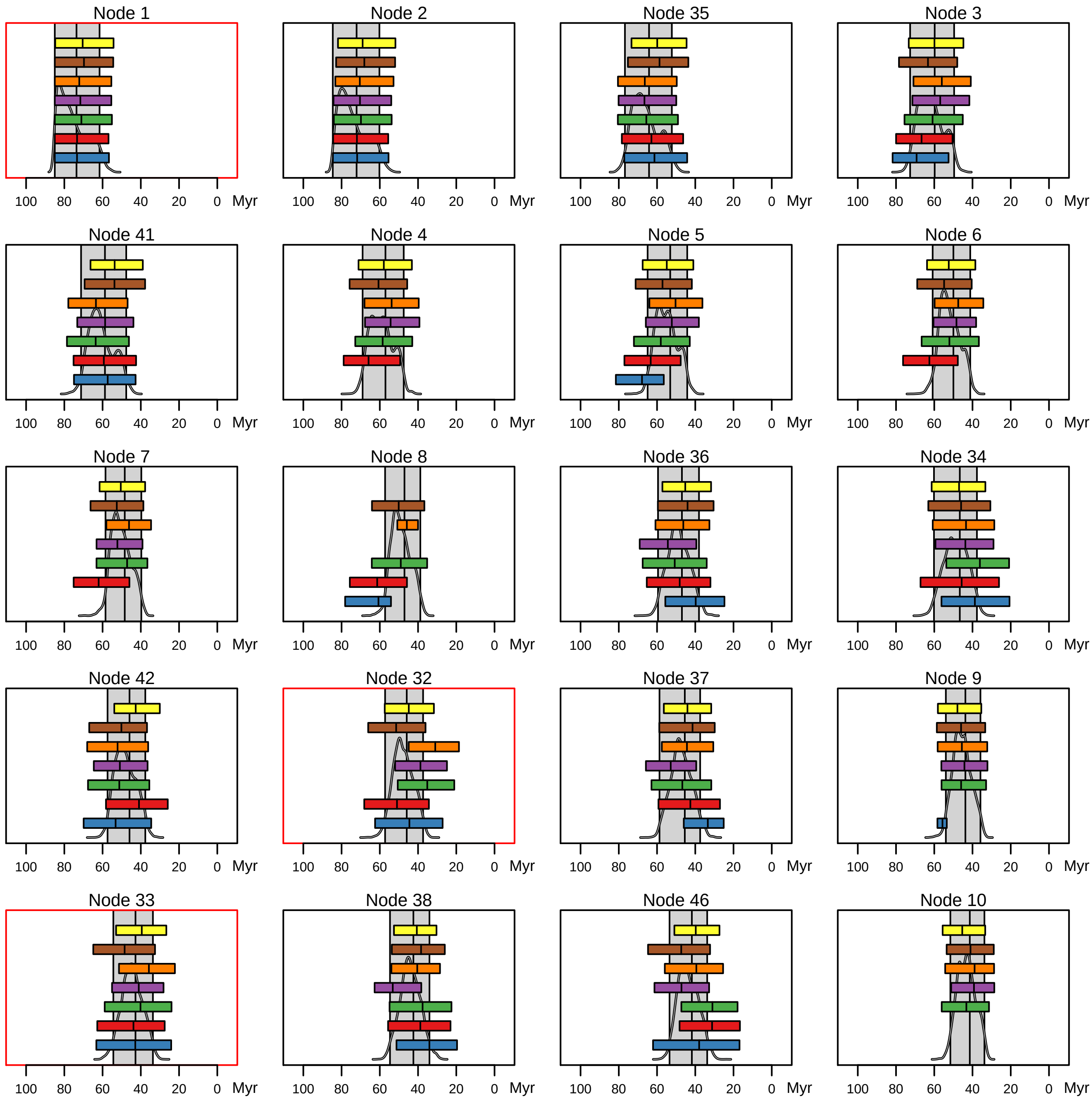
Node 1
Node 2
Node 35
Node 3
Node 41
Node 4
Node 5
Node 6
Node 7
Node 8
Node 36
Node 34
Node 42
Node 32
Node 37
Node 9
Node 33
Node 38
Node 46
Node 10
100 80 60 40 20 0 Myr

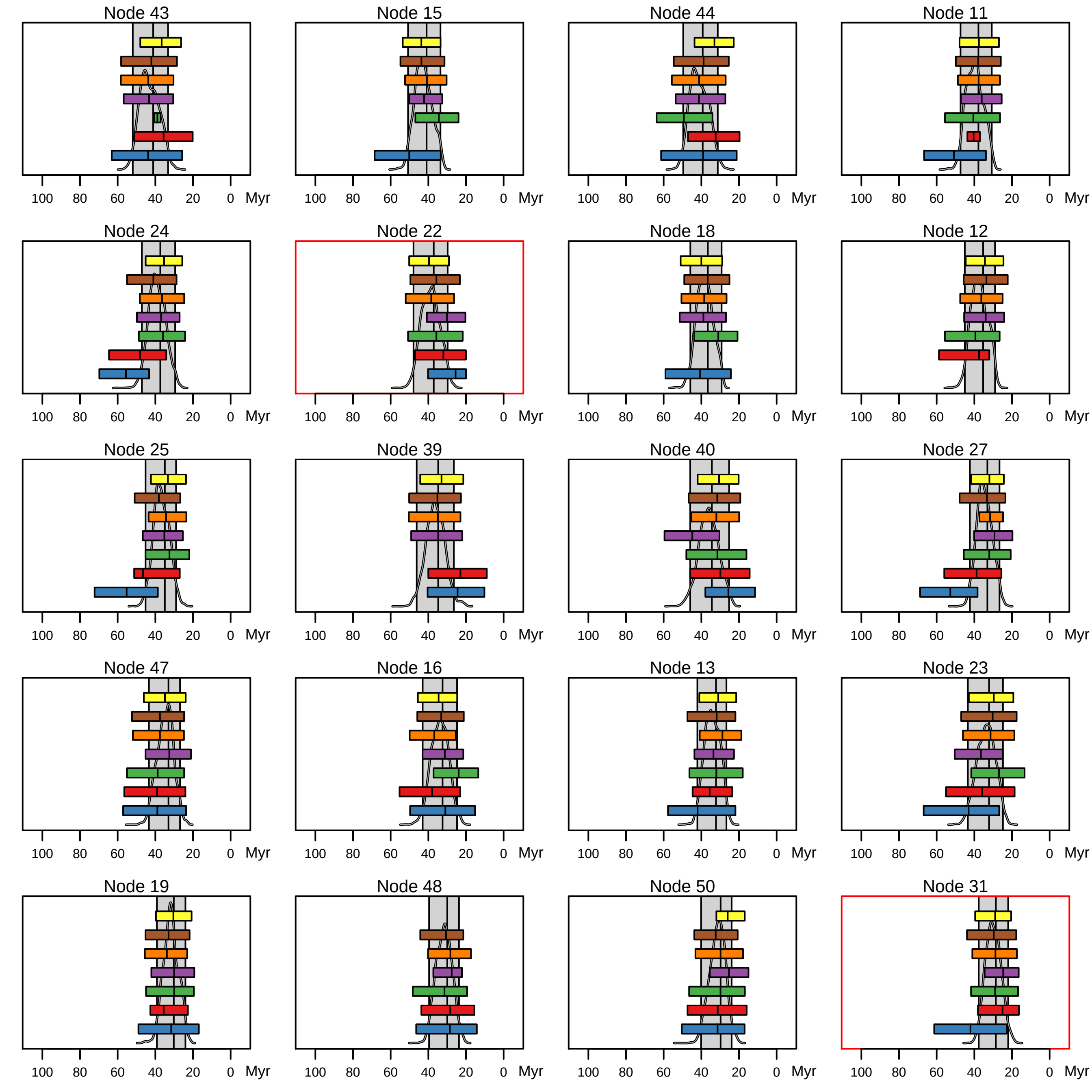
Node 43
Node 15
Node 44
Node 11
Node 24
Node 22
Node 18
Node 12
Node 25
Node 39
Node 40
Node 27
Node 47
Node 16
Node 13
Node 23
Node 19
Node 48
Node 50
Node 31
100 80 60 40 20 0 Myr

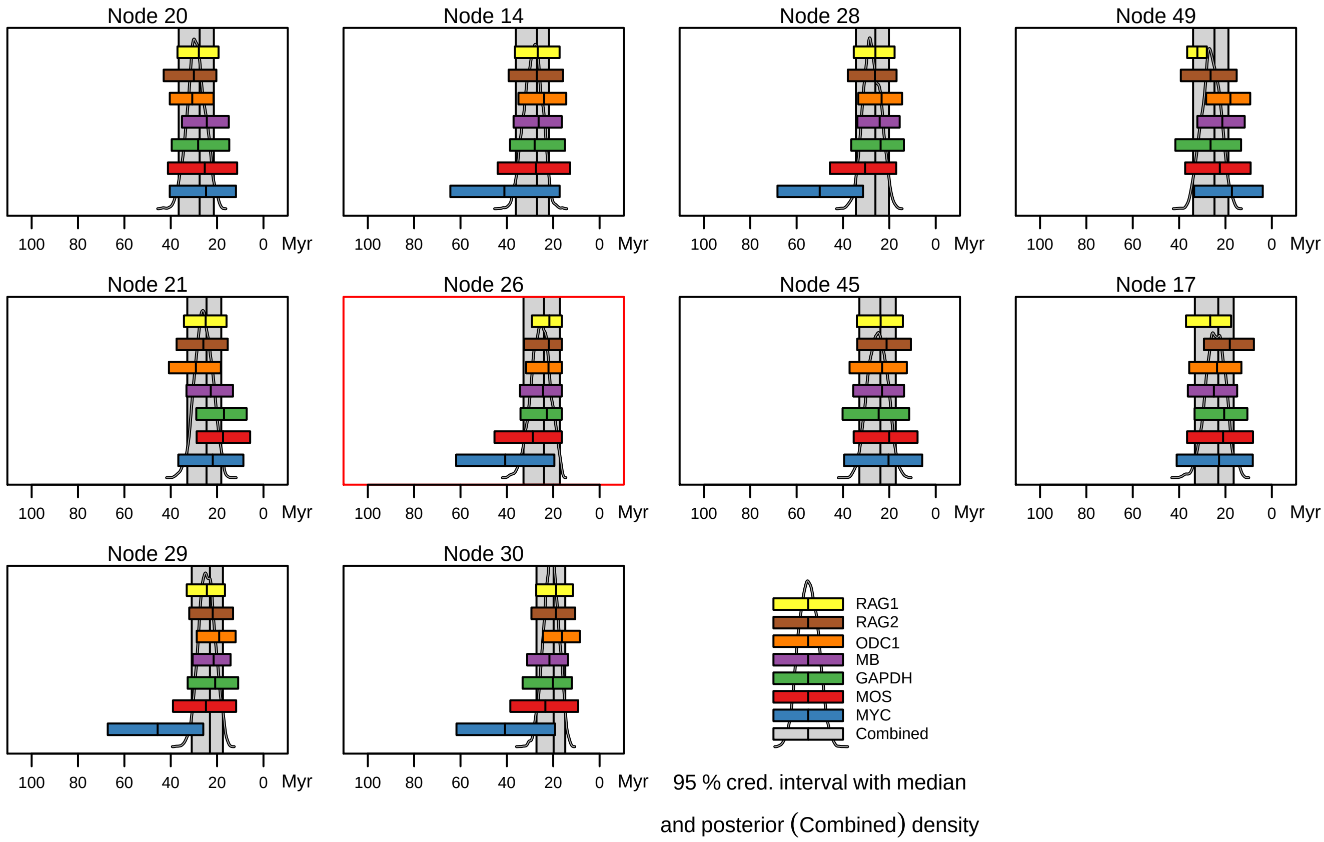

Node 20
Node 14
Node 28
Node 49
Node 21
Node 26
Node 45
Node 17
Node 29
Node 30
100 80 60 40 20 0 Myr
RAG1
RAG2
ODC1
MB
GAPDH
MOS
MYC
Combined
95 % cred. interval with median
and posterior (Combined) density
