# Supplementary material for: Dating the diversification of the major lineages of Passeriformes (Aves)
Source: BMC Evol Biol. 2014 Jan 15;14:8. doi: 10.1186/1471-2148-14-8 (PMC3917694; doi:10.1186/1471-2148-14-8)
Supplement: Additional file 6 — Text file S1. MrBayes command files including clock model settings and calibration information. [file 1471-2148-14-8-S6.docx]

**README file for MrBayes analysis**

# [Ericson et al. Dating the diversification of the major lineages of Passeriformes (Aves)]

# Run MrBayes v.3.2.2 with beagle library on Ericson et al. data

mb cmd.nex

**clock.nex -- command file for MrBayes v.3.2**

#NEXUS

[Ericson et al. Dating the diversification of the major lineages of Passeriformes (Aves)]

begin mrbayes;

[clock model settings, IGR (uncorrelated) clock model, root calibrated with Unif(52-85)]

prset brlenspr=clock:uniform;

prset clockvarpr=igr;

prset igrvarpr=exp(10.0);

prset clockratepr=lognorm(-7.386,1);

[calibration points]

constraint ingroup=2-.;

constraint A=1-.;

constraint B=Meliphagidae Malurus;

constraint C=Orthonyx Pomatostomus;

constraint D=Gymnorhina Batis;

constraint E=Oriolus Vireo;

constraint F=Sitta Troglodytes;

prset topologypr=constraints(ingroup,A,B,C,D,E,F);

prset nodeagepr=calibrated;

calibrate A=uniform(52,85);

calibrate B=offsetexp(10.4,58);

calibrate C=offsetexp(16.3,58);

calibrate D=offsetexp(16.3,58);

calibrate E=offsetexp(16.3,58);

calibrate F=offsetexp(20,58);

end;

**cmd.nex -- command file for MrBayes v.3.2**

#NEXUS

[Ericson et al. Dating the diversification of the major lineages of Passeriformes (Aves)]

begin mrbayes;

set autoclose=yes nowarn=yes;

log start filename=IGR.log replace;

exe data.nex;

exe model.nex;

exe clock.nex;

exe mcmcp.nex;

exe run.nex;

exe sum.nex;

log stop;

quit;

end;

**mcmcp.nex -- command file for MrBayes v.3.2**

#NEXUS

[Ericson et al. Dating the diversification of the major lineages of Passeriformes (Aves)]

begin mrbayes;

[mcmc]

set usebeagle=yes beagledevice=CPU beagleprecision=double beaglescaling=always beaglesse=no beagleopenmp=no;

[set usebeagle=no;]

mcmcp temp=0.1 nchain=4 samplefreq=1000 printfr=100 nruns=4 append=no;

mcmcp filename=IGR;

mcmcp ngen=30000000;

end;

**model.nex -- command file for MrBayes v.3.2**

#NEXUS

[Ericson et al. Dating the diversification of the major lineages of Passeriformes (Aves)]

begin mrbayes;

[partitioning]

charset CMOS_12 = 2-622\3 3-622\3;

charset CMOS_3 = 1-622\3;

charset CMYC_12 = 623-1126\3 624-1126\3;

charset CMYC_3 = 625-1126\3;

charset G3PDH = 1127-1545;

charset MYO = 1546-2345;

charset ODC = 2346-3094;

charset RAG1_12 = 3096-6041\3 3097-6041\3;

charset RAG1_3 = 3095-6041\3;

charset RAG2_12 = 6042-7193\3 6043-7193\3;

charset RAG2_3 = 6044-7193\3;

partition GeneCodon = 11: CMOS_12, CMOS_3, CMYC_12, CMYC_3, G3PDH, MYO, ODC, RAG1_12, RAG1_3, RAG2_12, RAG2_3;

set partition = GeneCodon;

[settings evol model]

lset applyto=(all) nucmodel=4by4 nst=mixed rates=invgamma covarion=no; [mixed+I+G]

unlink statefreq=(all) revmat=(all) shape=(all) pinvar=(all);

prset applyto=(all) ratepr=variable;

end;

**sum.nex -- command file for MrBayes v.3.2**

#NEXUS

[Ericson et al. Dating the diversification of the major lineages of Passeriformes (Aves)]

begin mrbayes;

sump burninfr=0.5 outputname=IGR;

sumt burninfr=0.5 outputname=IGR;

end;

**run.nex -- command file for MrBayes v.3.2**

#NEXUS

[Ericson et al. Dating the diversification of the major lineages of Passeriformes (Aves)]

begin mrbayes;

mcmc;

end;

**dat/data.nex -- data file**

[to be submitted]
